# The Lethality of [Pazopanib + HDAC Inhibitors] Is Enhanced by Neratinib

**DOI:** 10.3389/fonc.2019.00650

**Published:** 2019-07-18

**Authors:** Laurence Booth, Jane L. Roberts, Andrew Poklepovic, Paul Dent

**Affiliations:** ^1^Department of Biochemistry and Molecular Biology, Virginia Commonwealth University, Richmond, VA, United States; ^2^Department of Medicine, Virginia Commonwealth University, Richmond, VA, United States

**Keywords:** neratinib, pazopanib, entinostat, autophagy, HDAC inhibitor, crizotinib, chaperone, Hippo/YAP

## Abstract

Sarcomas are a diverse set of malignancies. For soft tissue sarcomas, the kinase and chaperone inhibitor pazopanib is a standard of care therapeutic. Previously, we demonstrated that HDAC inhibitors enhanced pazopanib lethality against sarcoma and other tumor cell types *in vitro* and *in vivo*. The present studies defined mechanisms of drug-combination resistance. Exposure of sarcoma and PDX ovarian carcinoma cells to [pazopanib + entinostat] caused a prolonged activation of ERBB1 and transient/prolonged activations of ERBB2, c-KIT, and c-MET, in a cell-specific fashion. The activities of mTORC1, mTORC2, GRP78, HSP90, and HSP70 were reduced, expression of Beclin1 and ATG5 enhanced, and the ATM-AMPK-ULK1-ATG13-Beclin1/ATG5 pathway activated. Inhibition of ERBB1/2/4 using neratinib or of c-MET using crizotinib significantly enhanced [pazopanib + entinostat] lethality. For neratinib with [pazopanib + entinostat], this effect correlated with reduced phosphorylation and expression of ERBB1, ERBB2, c-KIT, and c-MET and reduced expression, regardless of mutational status, of N-RAS and K-RAS. [Pazopanib + entinostat + neratinib] reduced the phosphorylation of the Hippo pathway proteins MST1/3/4 and MOB1 whereas this treatment increased the phosphorylation of LATS1, YAP, and TAZ. The activation of ATM, ULK-1, and eIF2α was further enhanced by [pazopanib + entinostat + neratinib] as was the expression of ATG5 and Beclin1. Compared to other manipulations, knock down of eIF2α or over-expression of BCL-XL significantly reduced killing by the three-drug interaction. *In vivo*, pazopanib and entinostat, and also neratinib and entinostat, both combined to significantly suppress the growth of sarcoma tumors.

## Introduction

Sarcomas are a rare and heterogeneous group of tumors with mesenchymal origin, accounting for ~1% of all human tumors. Sarcomas are categorized into two major groups according to primary tumor location: soft tissue sarcoma (STS) and bone sarcomas. The yearly incidence of STS cases in the United States is roughly 11,930, with an overall mortality of 4,870 deaths per year (1). Local control of STS can be obtained using surgery and radiotherapy. However, in ~30–40% of patients, disease will recur at distant sites, and of these, more than 90% ultimately will die from metastatic disease. In advanced and/or metastatic STS, the median overall survival is about 26 months with the current and most active combination chemotherapy regimens.

There are over 50 histologic subtypes of sarcoma, and as such, advances in therapeutic options have been slower to progress compared to other cancers. New agents and regimens are urgently needed to improve outcomes in this disease. Pazopanib is an FDA approved agent for most histologic subtypes of STS. This approval is based upon the randomized multicenter phase 3 “PALETTE” study, which evaluated pazopanib compared to placebo in 369 patients with STS that had progressed following frontline therapy (2). This study identified an improvement of overall survival of ~2 months (10.7–12.5 months) in patients treated with pazopanib. Despite demonstrating anti-tumor activity with a modest prolongation of survival, the best overall response for patients taking pazopanib was only partial response in 14 of 246 (6%). Given that this compound clearly prolongs survival but carries only a minimal anti-tumor response effect suggests that rationally designed combinations of pazopanib and other agents may improve outcomes.

The multi-kinase/chaperone inhibitor pazopanib is approved for the treatment of sarcoma and renal cell carcinoma. We previously determined that histone deacetylase inhibitors enhance the lethality of pazopanib in sarcoma cells and in PDX melanoma isolates (see NCT02795819) (3, 4). Follow-on studies next focused on the ability of HDAC inhibitors to alter tumor cell immunogenicity. Treatment of sarcoma cells with HDAC inhibitors reduced the expression of PD-L1, PD-L2, ornithine decarboxylase (ODC), and indoleamine-pyrrole 2,3-dioxygenase (IDO-1), and increased expression of the class I MHC protein MHCA. [Pazopanib + valproate] treated sarcoma cells released the highly immunogenic proteins HSP70 and HMGB1 into the extracellular environment (4, 5). HDAC inhibitors interacted with pazopanib to kill melanoma and sarcoma tumor cells *in vivo*.

We have recently published several manuscripts examining the biology of the irreversible ERBB1/2/4 inhibitor neratinib (6). Our most notable discovery was that not only did neratinib catalytically inhibit ERBB1/2/4, as it was designed to do, it also triggered the rapid internalization and subsequent proteolytic degradation of these receptors. As a negative control in our studies we had also examined the levels of the unrelated receptor c-MET. To our surprise, this receptor was also down-regulated following neratinib exposure, even though the receptor is not a neratinib target. One notable difference between ERBB1 and c-MET was that down-regulation of ERBB1 required a ubiquitination step, whereas c-MET did not. Growth factor receptors such as ERBB1/2/4 are often thought to exist on the surface of cells in quaternary complexes with other receptors and transducing proteins (7).

We also discovered that not only did neratinib cause ERBB1/2/4 and c-MET degradation, it was also capable of causing plasma membrane-associated mutant RAS proteins to be internalized and degraded (8–10). The ability of neratinib to trigger all these events were enhanced by HDAC inhibitors, which was attributed to inactivation of chaperone proteins and a more intense prolonged autophagic digestive process (11). Thus, in addition to inhibiting the primary evolution mechanism of compensatory survival signaling following [pazopanib + entinostat] exposure, neratinib could also attack other survival-related events such as RAS mutations. The present studies were designed to understand how neratinib could facilitate the lethality of [pazopanib + entinostat] and disrupt the evolution of primary resistance to the drug combination.

## Materials and Methods

### Materials

Pazopanib, crizotinib, sodium valproate, and entinostat were purchased from Selleckchem (Houston, TX). Neratinib was supplied by Puma Biotechnology Inc. (Los Angeles, CA). Cell culture materials were purchased from GIBCOBRL (GIBCOBRL Life Technologies, Grand Island, NY). The validated established cell lines: A498; HT1080; SKOV3; OVCAR4; MES-SA; SK-ES-1; and PANC1 were purchased from the ATCC. PAN02 cells were obtained from the NCI repository (Frederick, MD). UOK121LN and 786-0 cells were originally obtained from Dr. M. Lineman at the National Cancer Institute (Bethesda, MD. Spiky and N2 PDX ovarian cancer cells were obtained from Dr. Karen Paz Champions Oncology (Baltimore, MD, USA). Previously described PDX isolates of human head and neck cancer were kindly provided by Dr. John Lee (Chan Soon-Shiong Institute of Molecular Medicine, Culver City, CA, USA) (6, 8–10). Commercially available validated short hairpin RNA molecules to knock down RNA/protein levels were validated in house and purchased from Qiagen (Valencia, CA). Antibodies used: AIF (5318), BAX (5023), BAK (12105), BAD (9239), BIM (2933), BAK1 (12105), Beclin1 (3495), cathepsin B (31718), CD95 (8023), FADD (2782), eIF2α (5324), P-eIF2α S51 (3398), ULK-1 (8054), P-ULK-1 S757 (14202), P-AMPK S51 (2535), AMPKα (2532), P-ATM S1981 (13050), ATM (2873), ATG5 (12994), mTOR (2983), P-mTOR S2448 (5536), P-mTOR S2481 (2974), ATG13 (13468), MCL-1 (94296), BCL-XL (2764), P-AKT T308 (13038), P-ERK1/2 (5726), P-STAT3 Y705 (9145), P-p65 S536 (3033), p62 (23214), LAMP2 (49067) all from Cell Signaling Technology; P-ULK-1 S317 (3803a) from Abgent; P-ATG13 S318 (19127) from Novus Biologicals. The percentage knock down or over-expression of the specified proteins in HT1080 cells 24 h after transfection was: BAX, 83%; BAK, 86%; BID, 80%; BIM, 83%; NOXA, 84%; PUMA, 78%; ATM, 80%; AMPKα, 75%; ULK-1, 76%; mTOR, 83%; eIF2α, 85%; PERK, 90%; CD95, 81%; cathepsin B, 70%; BCL-XL, 79%; MCL-1, 84%; ATG5, 83%; Beclin1, 72%; AIF, 83%; ERBB1, 74%; ERBB2, 82%: ERBB3, 93%; ERBB4, 71%; CHOP, 83%; ATF4, 75%; ATF6, 79%; XBP-1, 76%; IRE1, 80%; dom. neg. caspase 9, 202%; SOD2, 234%; TRX, 230%; active MEK1, 269%; active AKT, 204%; active mTOR, 226%; BCL-XL, 295%; c-FLIP-s, 200%; dom. neg. IκB S32A S36A, 211%. Antibodies directed against RAS proteins: Thermo-Fisher (Waltham MA) N-RAS PA5-14833; K-RAS PA5-44339. We used two validated siRNA tools to knock down K-RAS or N-RAS for antibody validation purposes; custom made NRAS-5 CCUGAGUACUGACCUAAGAdTdT and K-RAS Silencer s7940. Knock down of K-RAS or N-RAS reduced fluorescent staining by ~80%. Methods of approach were as described (6, 8–10).

### Methods

#### Culture, Transfection, and *in vitro* Exposure of Cells to Drugs

All cell lines were cultured at 37°C (5% (v/v CO_2_) *in vitro* using RPMI supplemented with 5% (v/v) fetal calf serum and 10% (v/v) Non-essential amino acids. Cells were transfected with siRNA molecules or plasmids as described in prior manuscripts (6, 8–10). Cells were transfected with plasmids to express GFP-K-RAS V12 and RFP-K-RAS V12 (0.1 μg) using lipofectamine 2000. Twenty-four hours after transfection, cells were used in assays examining their staining for GFP and RFP.

#### Detection of Cell Viability, Protein Expression, and Protein Phosphorylation by Immuno-Fluorescence Using a Hermes WiScan Machine

http://www.idea-bio.com/ (6, 8–10). Trypan blue exclusion cell death analyses were performed using established protocols (6, 8–10). For immuno-fluorescence studies, cells were visualized in the Hermes system at either 10X or 60X. For all of the IF data, we set the machine, at 10X magnification, and the machine randomly determines the fluorescence intensity of ~120 cells per treatment condition. All immuno-fluorescent images for each individual protein/phospho-protein were taken using the identical machine settings. Images were processed at 9,999 dpi and at 32 bit using Adobe Photoshop, and figures labeled in Microsoft PowerPoint.

#### Animal Studies

Studies were performed per USDA regulations under VCU IACUC protocol AD20008. HT1080 STS cells (2 ×10^6^) were implanted into the rear right flanks of male NRG mice. Tumors were permitted to growth unchallenged until their volume was ~50 mm^3^. Animals were treated for 7 days with vehicle control (VEH, cremophore QD), entinostat (ENT, 1 mg/kg, Q Day 1 only), pazopanib (PAZ, 20 mg/kg QD), neratinib (NER, 10 mg/kg QD), or the drugs in combination as indicated (*n* = 10 mice per group). The -fold increase in tumor mass/volume compared to the mass at Day 0 (defined as 1.00) is plotted (±SEM).

#### Data Analysis

Comparison of the effects of various treatments (in triplicate three times) was using one-way ANOVA and a two tailed Student's *t*-test. Statistical examination of *in vivo* animal survival data utilized a two tailed Student's *t*-test and log rank statistical analyses between the different treatment groups. Differences with a *p* < 0.05 were considered statistically significant. Experiments are the means of multiple individual points from multiple experiments (± SEM).

## Results

**Figure 1:**
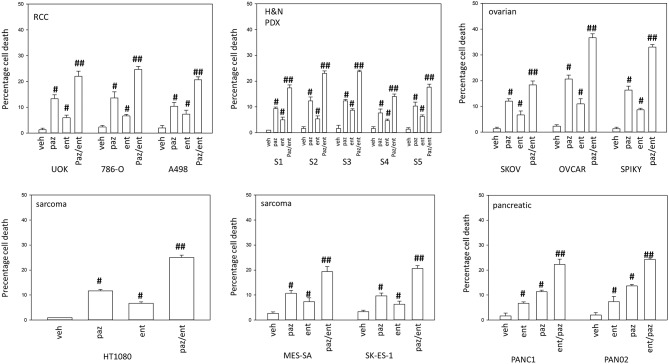
Entinostat enhances pazopanib lethality in a genetically diverse set of tumor cells. Sarcoma (HT1080, MES, SKE); renal carcinoma (UOK121LN, A498); squamous head & neck carcinoma (S1, S2, S3, S4, S5); ovarian carcinoma (SKOV3, OVCAR4, Spiky); pancreatic (PANC-1, PAN02) were treated with vehicle control (VEH), pazopanib (PAZ, 1.0 μM), entinostat (ENT, 50 nM) or the drugs in combination for 24 h. Cells were isolated, and viability determined by trypan blue exclusion (*n* = 3 ± SEM). ^#^*p* < 0.05 greater than vehicle control; ^##^*p* < 0.05 greater than pazopanib alone value.

**Figure 2:**
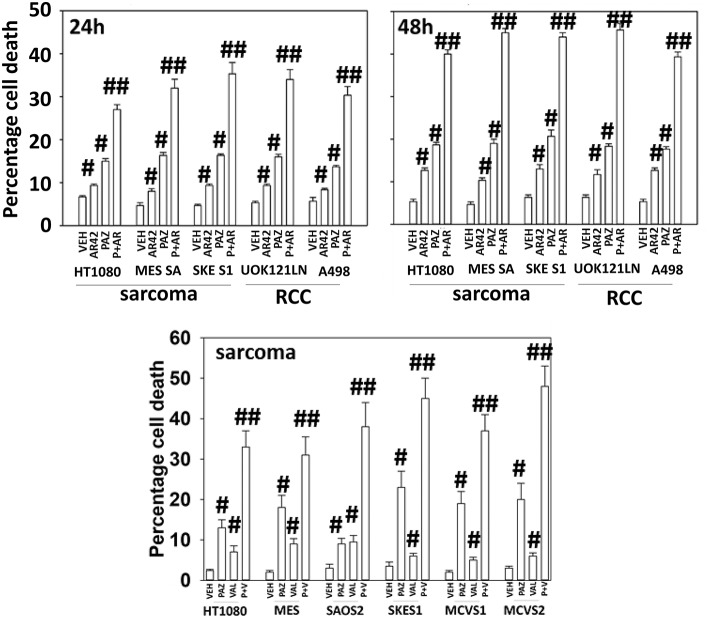
HDAC inhibitors enhance pazopanib lethality in renal and sarcoma cells. Sarcoma (HT1080, MES, SKE, SAOS2, PDX MCVS1, PDX MCVS2) and renal carcinoma cells (UOK121LN, A498) were treated with vehicle control (VEH), pazopanib (PAZ, 1.0 μM), AR42 (600 nM), sodium valproate (VAL, 500 nM) or the drugs in combination for 24 and 48 h as shown in the graphical panels. Cells were isolated, and viability determined by trypan blue exclusion (*n* = 3 ± SEM). ^#^*p* < 0.05 greater than vehicle control; ^##^*p* < 0.05 greater than pazopanib alone value.

Pazopanib interacted with the HDAC inhibitor entinostat to kill ovarian, sarcoma, renal, head and neck, and pancreatic cancer cells (Figure 1). In sarcoma and renal carcinoma cells, pazopanib interacted in a greater than additive fashion with the HDAC inhibitors sodium valproate and AR42 to cause cell death (Figure 2). Prior work had shown pazopanib also interacted with the HDAC inhibitor vorinostat to kill tumor cells (3). Thus, the concept of combining pazopanib with HDAC inhibitors to achieve enhanced killing appears to occur regardless of tumor cell type or HDAC inhibitor chemistry.

**Figure 3:**
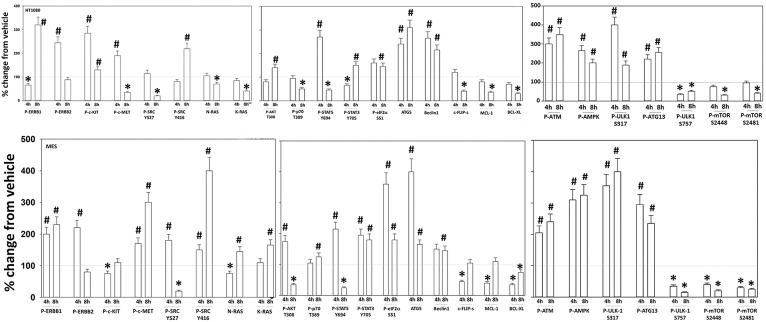
Regulation of signaling by [pazopanib + entinostat] in human sarcoma cells. Human sarcoma cells were treated with vehicle control or with [pazopanib (1.0 μM) + entinostat (50 nM)] for 4 or 8 h. At each time point cells were fixed in place and immunofluorescence staining performed to detect the total expression and phosphorylation of the indicated proteins under vehicle control and drug exposed conditions. The percentage change in fluorescence intensity compared to vehicle control, defined as 100% is plotted; this value is further normalized based on the total expression of each protein under each condition, i.e., stochiometric phosphorylation (*n* = 3 ± SEM). ^*^p < 0.05 less than control value; ^#^*p* < 0.05 greater than control value.

**Figure 4:**
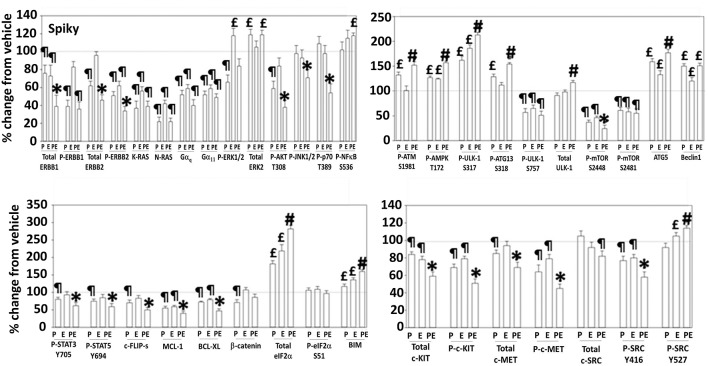
Regulation of signaling by [pazopanib + entinostat] in Spiky human PDX ovarian carcinoma cells. PDX human ovarian carcinoma cells were treated with vehicle control, pazopanib (1.0 μM), entinostat (50 nM) or the drugs in combination for 6 h. At each time point cells were fixed in place and immunofluorescence staining performed to detect the expression and phosphorylation of the indicated proteins under vehicle control and drug exposed conditions. The percentage change in fluorescence intensity compared to vehicle control, defined as 100% is plotted (*n* = 3 ± SEM). ^¶^*p* < 0.05 less than vehicle control; ^*^p < 0.05 less than pazopanib; ^£^*p* < 0.05 greater than vehicle control value; ^#^*p* < 0.05 greater than [P+E] value.

**Figure 5:**
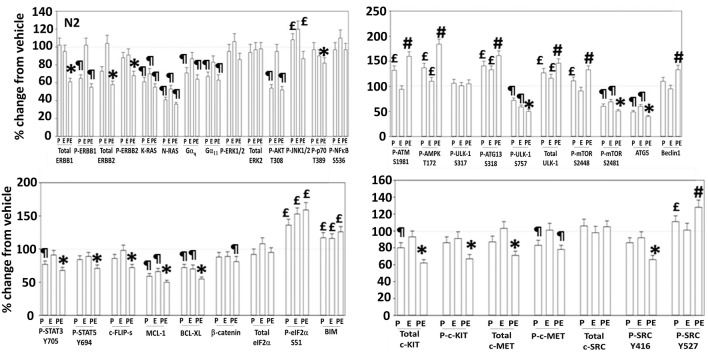
Regulation of signaling by [pazopanib + entinostat] in N2 human PDX ovarian carcinoma cells. PDX human ovarian carcinoma cells were treated with vehicle control, pazopanib (1.0 μM), entinostat (50 nM) or the drugs in combination for 6 h. At each time point cells were fixed in place and immunofluorescence staining performed to detect the expression and phosphorylation of the indicated proteins under vehicle control and drug exposed conditions. The percentage change in fluorescence intensity compared to vehicle control, defined as 100% is plotted (*n* = 3 ± SEM). ^¶^*p* < 0.05 less than vehicle control; ^*^p < 0.05 less than pazopanib; ^£^*p* < 0.05 greater than vehicle control value; ^#^*p* < 0.05 greater than pazopanib value.

Additional studies then determined the impact of [pazopanib + entinostat] exposure on cell signaling in sarcoma and ovarian carcinoma cells; pazopanib is FDA approved for the treatment of sarcoma and is compendium listed for the treatment of ovarian carcinoma. Exposure of sarcoma cells to [pazopanib + entinostat] caused activation of ERBB1 and c-SRC and in a cell-specific fashion, activations of ERBB2, c-KIT, and c-MET (Figure 3). In PDX isolates of ovarian cancer cells, however, the expression and phosphorylation of ERBB1, ERBB2, c-MET, c-KIT, and c-SRC was reduced by drug combination exposure (Figures 4, 5). The activities of mTORC1 and mTORC2 were reduced, expression of Beclin1 and ATG5 enhanced, and the ATM-AMPK-ULK1-ATG13-Beclin1/ATG5 pathway activated. Endoplasmic reticulum (ER) stress signaling was increased in sarcoma and ovarian cancer cells as judged by elevated PERK and eIF2α phosphorylation, whereas only in sarcoma cells was drug-induced activation of SRC observed.

**Figure 6:**
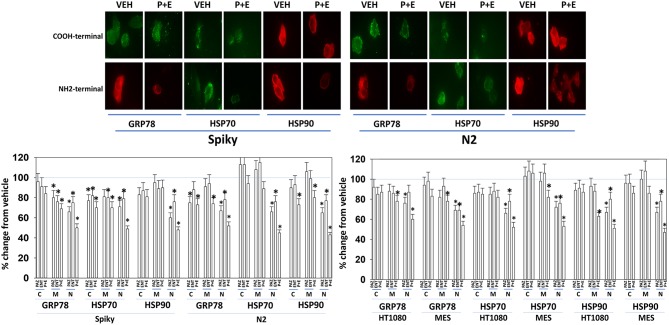
Pazopanib and entinostat combine to reduce the NH2-terminal immuno-reactivity of GRP78, HSP70, and HSP90. Sarcoma (HT1080, MES) and PDX ovarian carcinoma (Spiky, N2) cells were treated with vehicle control (VEH), pazopanib (PAZ, 1.0 μM), entinostat (ENT, 50 nM) or the drugs in combination for 6 h. At each time point cells were fixed in place and immunofluorescence staining performed to detect epitopes located at the NH2-terminus, the COOH-terminus, and an epitope located in approximately the middle of the protein. Based on the commercially antibodies available and based on their relative abilities to stain the chaperones, we utilized antibodies from mouse, rabbit, and goat and chosen secondary red or green fluorescent secondary antibodies for detection. The percentage change in fluorescence intensity compared to vehicle control, defined as 100% is plotted (*n* = 3 ± SEM). ^*^p < 0.05 less than vehicle control value. Upper IF images: representative images of changes in the fluorescent staining for the COOH-terminus and the NH2-terminus of GRP78, HSP70, and HSP90 in PDX ovarian carcinoma cells.

Several years ago, we published data showing that pazopanib could act as a low-affinity inhibitor of multiple chaperone proteins (12). Pazopanib caused a conformational change in the NH2-termini of chaperones such that epitopes were hidden from antibody detection and is where the chaperones bind and hydrolyze ATP as part of their function but did not alter antibody staining for the COOH termini. In our present studies, pazopanib reduced the NH2-terminal IF staining intensity for GRP78, HSP90, and HSP70 but did not alter antibody staining levels directed at the COOH termini (Figure 6). In all cells tested, entinostat enhanced the ability of pazopanib to reduce the staining intensities of the chaperone NH2-termini. Reduced chaperone function correlates with the data in Figure 2 showing that endoplasmic reticulum stress was being induced.

**Figure 7:**
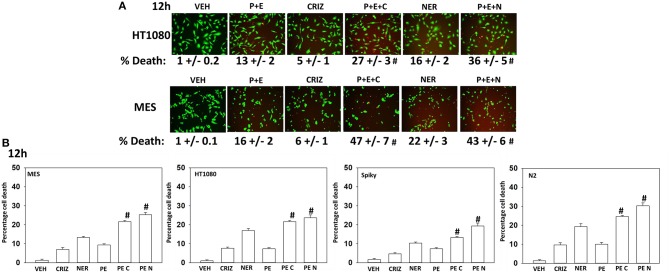
[Pazopanib + entinostat] lethality is enhanced by the c-MET inhibitor crizotinib or by the irreversible ERBB1/2/4 inhibitor neratinib. **(A)** Human sarcoma cells were treated with vehicle control (VEH), [pazopanib (PAZ, 1.0 μM) + entinostat (ENT, 50 nM)], crizotinib (CRIZ, 0.5 μM), neratinib (NER, 50 nM) or the drugs as indicated in combination for 12 h. Cells were isolated, and viability determined by live/dead assays (n = 3 ± SEM). ^#^*p* < 0.05 greater than corresponding [P+E] value. **(B)** Human sarcoma and PDX ovarian carcinoma cells were treated with vehicle control, [pazopanib (1.0 μM) + entinostat (50 nM)], crizotinib (0.5 μM), neratinib (50 nM) or the drugs as indicated in combination for 12 h. Cells were isolated, and viability determined by trypan blue exclusion (*n* = 3 ± SEM). ^#^*p* < 0.05 greater than corresponding [P+E] value.

**Figure 8:**
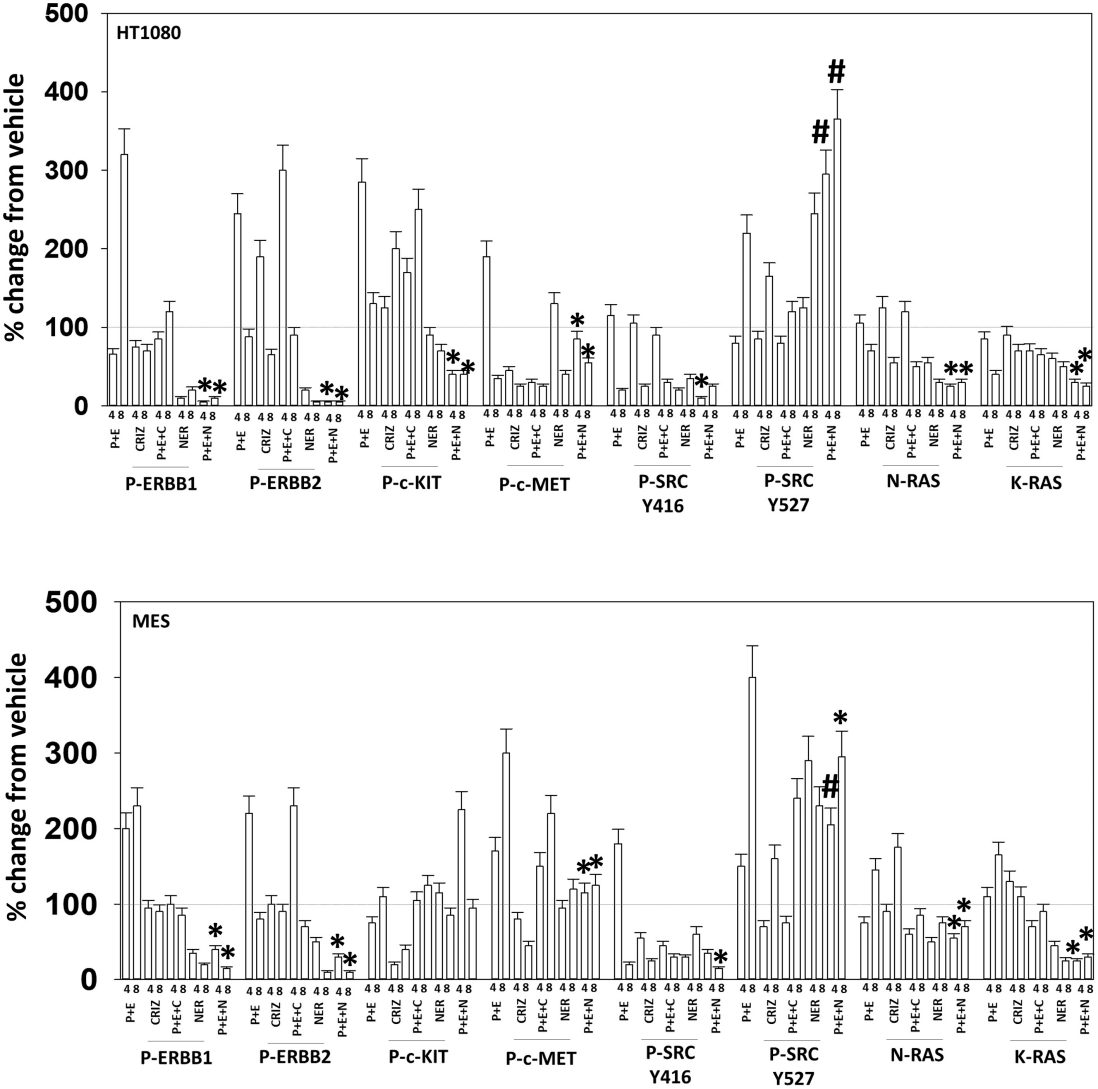
Neratinib blocks compensatory survival signaling after exposure of HT1080 and MES cells to [pazopanib + entinostat]. Human sarcoma cells were treated with vehicle control (VEH), [PAZ, pazopanib (1.0 μM) + entinostat (ENT, 50 nM)], crizotinib (CRIZ, 0.5 μM), neratinib (NER, 50 nM) or the drugs as indicated in combination for 4 and 8 h. At each time point cells were fixed in place and immunofluorescence staining performed to detect the expression and phosphorylation of the indicated proteins. The percentage change in fluorescence intensity compared to vehicle control (defined as 100%) is plotted (*n* = 3 ± SEM). ^*^p < 0.05 less than [P+E] value; ^#^*p* < 0.05 greater than [P+E] value.

**Figure 9:**
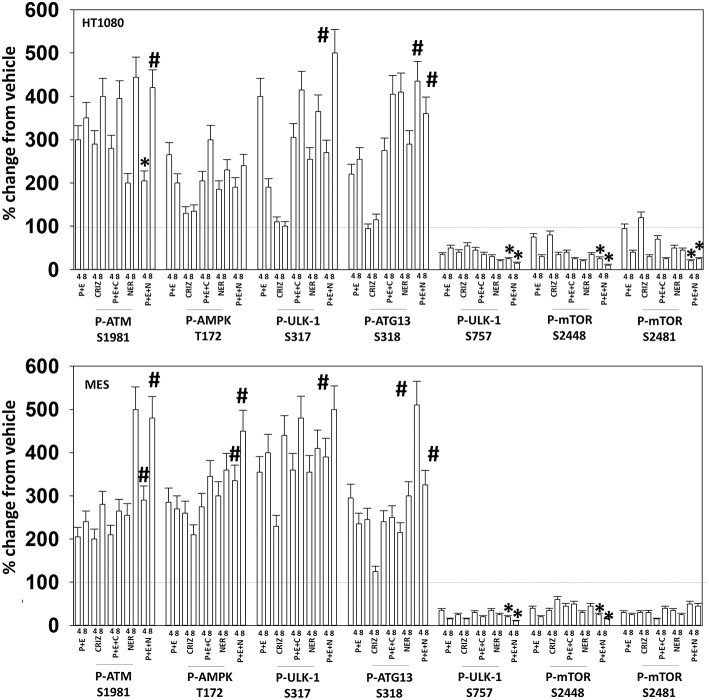
Neratinib blocks compensatory survival signaling after exposure of MES and HT1080 cells to [pazopanib + entinostat]. Human sarcoma cells were treated with vehicle control (VEH), [PAZ, pazopanib (1.0 μM) + entinostat (ENT, 50 nM)], crizotinib (CRIZ, 0.5 μM), neratinib (NER, 50 nM) or the drugs as indicated in combination for 4 and 8 h. At each time point cells were fixed in place and immunofluorescence staining performed to detect the expression and phosphorylation of the indicated proteins. The percentage change in fluorescence intensity compared to vehicle control (defined as 100%) is plotted (*n* = 3 ± SEM). ^*^p < 0.05 less than [P+E] value; ^#^*p* < 0.05 greater than [P+E] value.

**Figure 10:**
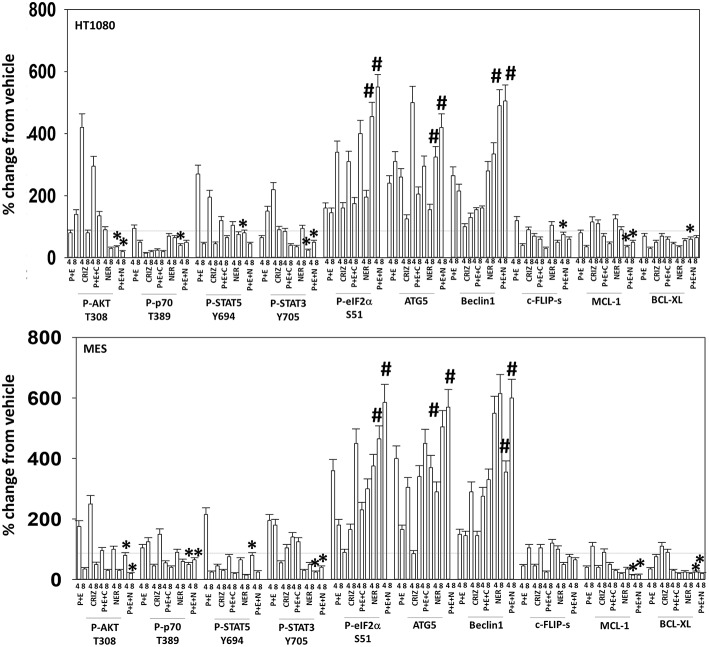
Neratinib blocks compensatory survival signaling after exposure of human sarcoma cells to [pazopanib + entinostat]. Human sarcoma cells (HT1080, MES) were treated with vehicle control (VEH), [PAZ, pazopanib (1.0 μM) + entinostat (ENT, 50 nM)], crizotinib (CRIZ, 0.5 μM), neratinib (NER, 50 nM) or the drugs as indicated in combination for 4 and 8 h. At each time point cells were fixed in place and immunofluorescence staining performed to detect the expression and phosphorylation of the indicated proteins. The percentage change in fluorescence intensity compared to vehicle control (defined as 100%) is plotted (*n* = 3 ± SEM). ^*^p < 0.05 less than [P+E] value; ^#^*p* < 0.05 greater than [P+E] value.

Based on the receptor phosphorylation data in Figures 3–5, we next examined whether inhibitors of ERBB1/2/4 (neratinib) or of c-MET (crizotinib) could enhance the lethality of [pazopanib + entinostat]. As assessed by live/dead and trypan blue exclusion viability assays, both neratinib and crizotinib significantly enhanced the lethality of [pazopanib + entinostat] (Figures 7A,B). We then re-examined changes in cell signaling for [pazopanib + entinostat] alone or in combination with either neratinib or crizotinib. Compared to crizotinib, neratinib, overall, more effectively suppressed compensatory “survival signaling” pathways that were induced following [pazopanib + entinostat] exposure (Figures 8–10). In the presence of neratinib, the basal or stimulated activities of ERBB1, ERBB2, c-KIT, and c-MET were prevented. Neratinib reduced SRC activation and caused down-regulation of N-RAS and K-RAS. Neratinib combined with [pazopanib + entinostat] promoted greater activation of ATM, ULK-1 and increased ATG13 S318 phosphorylation whereas it caused enhanced inactivation of AKT, STAT3, and STAT5. These events would collectively be assumed to promote greater levels of autophagosome formation, in parallel with reduced protective signaling via AKT and STAT transcription factors. The combination facilitated a further reduction in the expression of the cyto-protective proteins c-FLIP-s, BCL-XL, and MCL-1 and acted to enhance levels of the autophagy regulatory proteins Beclin1 and ATG5. In cells exposed to [pazopanib + entinostat + neratinib] the activities of ERK1/2, JNK1/2, and p38 MAPK also all declined (data not shown).

**Figure 11:**
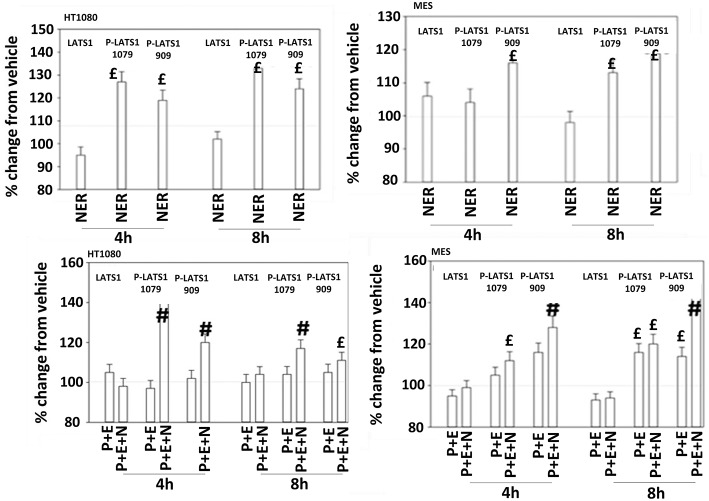
Neratinib, and when combined with [pazopanib + entinostat] activates LATS1 in HT1080 and MES cells. Human sarcoma cells were treated with vehicle control, [pazopanib (P, 1.0 μM) + entinostat (E, 50 nM)], neratinib (N, 50 nM) or the drugs as indicated in combination for 4 and 8 h. At each time point cells were fixed in place and immunofluorescence staining performed to detect the expression and phosphorylation of the indicated proteins. The percentage change in fluorescence intensity compared to vehicle control (defined as 100%) is plotted (*n* = 3 ± SEM).^£^*p* < 0.05 greater than vehicle control value; ^#^*p* < 0.05 greater than [P+E] value.

**Figure 12:**
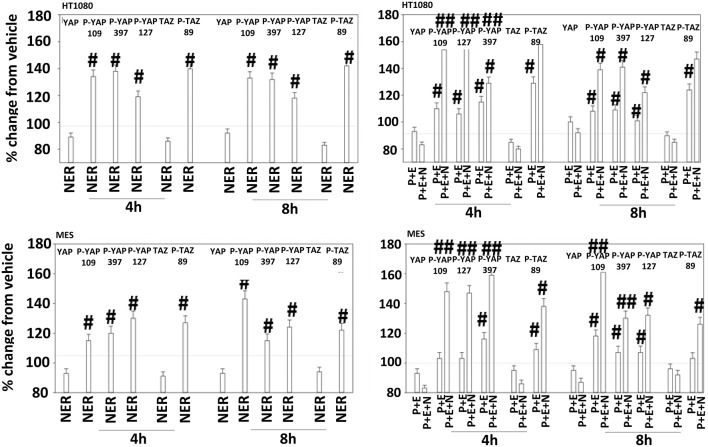
Neratinib, and when combined with [pazopanib + entinostat] reduces YAP/TAZ function in HT1080 and MES cells. Human sarcoma cells were treated with vehicle control (VEH), [pazopanib (P, 1.0 μM) + entinostat (E, 50 nM)], neratinib (N, 50 nM) or the drugs as indicated in combination for 4 and 8 h. At each time point cells were fixed in place and immunofluorescence staining performed to detect the expression and phosphorylation of the indicated proteins. The percentage change in fluorescence intensity compared to vehicle control (defined as 100%) is plotted (*n* = 3 ± SEM). ^#^*p* < 0.05 greater than vehicle control value; ^##^*p* < 0.05 greater than corresponding [P+E] value.

The irreversible inhibitor neratinib was developed to inhibit ERBB1/2/4 at nanomolar concentrations. It was then discovered that the drug is equipotent at inhibiting multiple Ste20 family kinases especially MST3, MST4, MAP4K5, and MAP4K3 (13). MST3/4 control the apical brush border of epithelial cells; the major dose limiting toxicity of neratinib is diarrhea, arguing that neratinib is acting in an on-target fashion to cause this event. MST3/4 coordinate the phosphorylation of cytoskeletal proteins such as Ezrin/Radixin/Moesin (ERM) family to regulate plasma membrane ruffling (14–18). MAP4K5 is an apical kinase that interacts with GTP binding proteins downstream of G Protein Coupled Receptors, and links GPCR signaling into MAPK pathways. MAP4K5 and MAP4K3 phosphorylate and activate the LATS1/2 kinases that in turn phosphorylate and inhibit YAP/TAZ, the main effectors of the Hippo pathway (19–21). MAP4K3 has also been shown to play an important role in amino acid signaling to mTOR/p70 S6K. Treatment of cells with neratinib modestly reduced the phosphorylation of MOB1, MST1, MST3, and MST4 (Figures S1, S2). Exposure to [pazopanib + entinostat + neratinib] did not significantly enhance dephosphorylation of these enzymes. Similar data were obtained examining the phosphorylation of LATS1 (Figure 11). In contrast, whilst neratinib enhanced the phosphorylation of TAZ and at multiple sites in YAP, exposure to the three-drug combination significantly further enhanced TAZ and YAP phosphorylation (Figure 12). These data argue that [pazopanib + entinostat] treatment modulates TAZ and YAP function directly, and not via canonical MST-LATS signaling.

**Figure 13:**
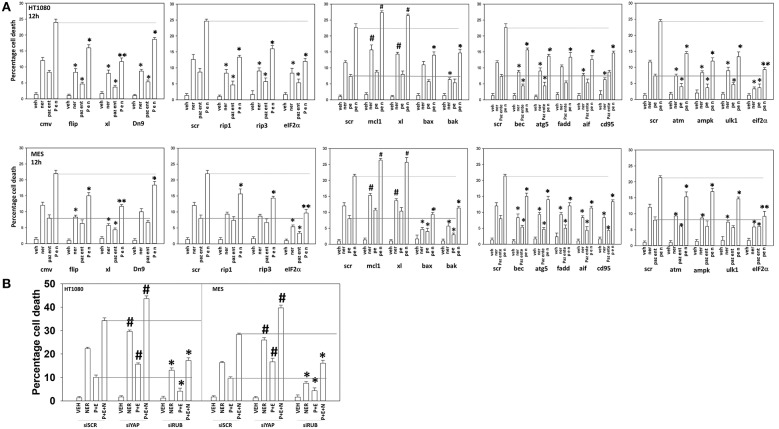
Neratinib interacts with [pazopanib + entinostat] through ER stress and mitochondrial dysfunction to kill sarcoma cells *in vitro* and *in vivo*. **(A)** Human sarcoma cells were transfected with an empty vector plasmid (CMV) or with a scrambled siRNA or cells were transfected with plasmids to express BXL-XL, c-FLIP-s, or dominant negative caspase 9, or with siRNA molecules to knock down expression of the indicated proteins. Twenty-four hour after transfection, cells were treated with vehicle control (VEH), neratinib (NER, 50 nM), [pazopanib (P, 1.0 μM) + entinostat (E, 50 nM)] or [pazopanib + entinostat + neratinib] in combination for 12 h. Cell viability was determined by trypan blue exclusion assay (*n* = 3 ± SEM). ^*^p < 0.05 less than corresponding value in control transfected cells; ^**^p < 0.01 less than corresponding value in control transfected cells; ^#^*p* < 0.05 greater than corresponding value in control transfected cells. **(B)** Human sarcoma cells were transfected with a scrambled siRNA or with siRNA molecules to knock down expression of YAP or Rubicon. Twenty-four hour after transfection, cells were treated with vehicle control (VEH), neratinib (NER, 50 nM), [pazopanib (P, 1.0 μM) + entinostat (E, 50 nM)], or [pazopanib + entinostat + neratinib] in combination for 12 h. Cell viability was determined by trypan blue exclusion assay (*n* = 3 ± SEM). ^*^p < 0.05 less than corresponding value in control transfected cells; ^#^*p* < 0.05 greater than corresponding value in control transfected cells.

We next defined the survival-regulatory pathways by which neratinib enhanced [pazopanib + entinostat] lethality. As a single agent, the lethality of neratinib was reduced, as previously observed, by preventing induction of the ATM-AMPK-autophagy pathway and endoplasmic reticulum signaling (6) (Figure 13A). Over-expression of BCL-XL, to a significantly greater extent than expression of dominant negative caspase 9 or over-expression of c-FLIP-s, reduced the lethality of the three-drug combination. The requirement of ER stress signaling for the induction of death was significantly more essential to the killing process than was knock down of autophagy-regulatory or death receptor-regulatory proteins. Knock down of apoptosis inducing factor (AIF), i.e., necroptotic death, was significantly more protective than expression of dominant negative caspase 9.

**Figure 14:**
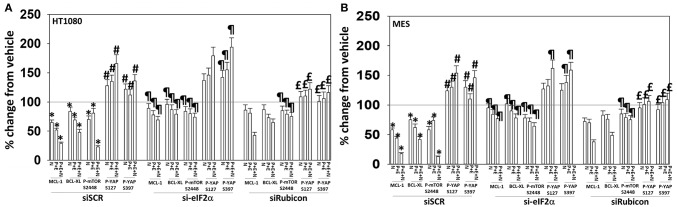
Knock down of eIF2α prevents the reduction in MCL-1/BCL-XL expression and knock down of Rubicon prevents phosphorylation of YAP. **(A)** HT1080 and MES cells were transfected with a scrambled control siRNA (siSCR) or with an siRNA to knock down expression of eIF2α. Twenty-four hours after transfection, cells were treated with vehicle control (VEH), [pazopanib (P, 1.0 μM) + entinostat (E, 50 nM)], neratinib (N, 50 nM) or the drugs as indicated in combination for 6 h. Cells were fixed in place and immunofluorescence staining performed to detect the expression and phosphorylation of the indicated proteins. The percentage change in fluorescence intensity compared to vehicle control, defined as 100%, is plotted (*n* = 3 ± SEM). ^*^p < 0.05 less than vehicle control value; ^#^*p* < 0.05 greater than vehicle control value; ^¶^*p* < 0.05 greater than corresponding value in siSCR cells; ^£^*p* < 0.05 less than corresponding value in siSCR cells. **(B)** HT1080 and MES cells were transfected with a scrambled control siRNA (siSCR) or with an siRNA to knock down expression of Rubicon. Twenty-four hour after transfection, cells were treated with vehicle control, [pazopanib (1.0 μM) + entinostat (50 nM)], neratinib (50 nM) or the drugs as indicated in combination for 6 h. Cells were fixed in place and immunofluorescence staining performed to detect the expression and phosphorylation of the indicated proteins. The percentage change in fluorescence intensity compared to vehicle control, defined as 100%, is plotted (*n* = 3 ± SEM). ^*^p < 0.05 less than vehicle control value; ^#^*p* < 0.05 greater than vehicle control value; ^¶^*p* < 0.05 greater than corresponding value in siSCR cells; ^£^*p* < 0.05 less than corresponding value in siSCR cells.

We then determined that knock down of YAP enhanced the lethality of neratinib, [pazopanib + entinostat] and the three-drug combination (Figure 13B). We have published that treatment of cells with either [pazopanib + HDAC inhibitor] or [neratinib + HDAC inhibitor] increases the levels of autophagosomes and autolysosomes in tumor cells, and that knock down of the autophagy regulatory proteins Beclin1 or ATG5 significantly suppressed autophagy and tumor cell killing. More recently, we have shown that neratinib is capable of inducing LC3-associated phagocytosis (LAP), which requires expression of the protein Rubicon; knock down of Rubicon was significantly more protective than knock down of Beclin1 or ATG5 at preventing tumor cell killing (Figure 13B) (22). Knock down of Rubicon was significantly better at preventing tumor cell killing compared to knock down of Beclin1 (Figures 14A,B). Knock down of eiF2α dampened the drug-induced inactivation of mTOR and further enhanced phosphorylation of YAP. Knock down of eIF2α prevented drug exposure from reducing MCL-1 or BCL-XL expression. Knock down of Rubicon prevented both mTOR inactivation and YAP phosphorylation but did not prevent MCL-1 and BCL-XL down-regulation.

**Figure 15:**
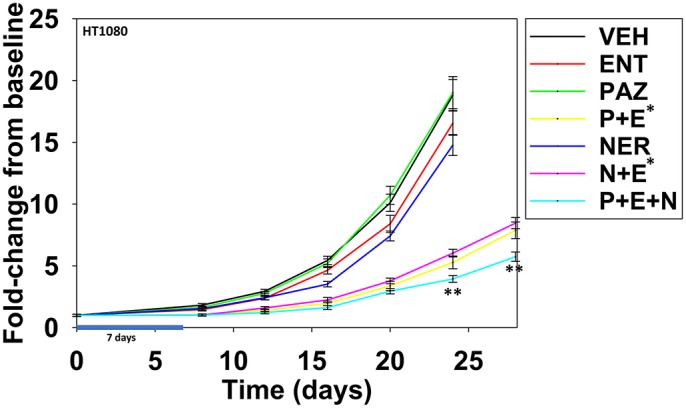
Entinostat enhances the lethality of pazopanib and of neratinib against HT1080 fibrosarcoma tumors. Male NRG mice were implanted with 2 ×10^6^ HT1080 cells in their rear right flank and after seven days tumors of ~50 mm^3^ had grown. Animals were treated for 7 days with vehicle control (VEH); neratinib (NER, 10 mg/kg, QD); entinostat (ENT, 1 mg/kg, Q day 1); pazopanib (PAZ, 20 mg/kg, QD); [pazopanib + entinostat]; [neratinib + entinostat]; or [pazopanib + entinostat + neratinib]. Initial tumor volume for vehicle control 52.0 mm^3^; initial tumor volume for neratinib 50.2 mm^3^; initial tumor value for pazopanib 45.4 mm^3^; initial tumor volume for entinostat 49.9 mm^3^; initial tumor volume for [P+E] 56.8 mm^3^; initial tumor volume for [N+E] 58.4 mm^3^; initial tumor volume for [P+E+N] 61.3 mm^3^. Tumor volumes were measured every 3–4 days until tumor volumes were >1,500 mm^3^ (*n* = 10 per group ±SEM). ^*^p < 0.05 less than entinostat alone; ^**^p < 0.05 less than corresponding time points in [pazopanib + entinostat]; or [neratinib + entinostat].

Finally, we determined whether neratinib could safely enhance the anti-tumor efficacy of [pazopanib + entinostat] *in vivo* against the HT1080 human fibrosarcoma cell line. Animals were dosed, alone or in combination, continuously for seven days. Neratinib was dosed at ~20% of its safe plasma C max; pazopanib was dosed at ~10% of its safe plasma C max; entinostat was dosed once and based on patient data at 1 mg/kg which is ~75% of its C max value (19). Pazopanib and entinostat interacted to significantly further reduce the growth of sarcoma tumors below that of either individual agent (Figure 15; *p* < 0.05). Neratinib and entinostat interacted to significantly further reduce the growth of sarcoma tumors below that of either individual agent (*p* < 0.05). At later time points, neratinib significantly, though modestly, enhanced the anti-tumor efficacy of [pazopanib + entinostat] (*p* < 0.05).

## Discussion

The present studies were designed to build upon our prior studies combining pazopanib with other HDAC inhibitors, which resulted in the phase I clinical trial NCT02795819. As the experimental HDAC inhibitor AR42 is no-longer available for clinical use, we realized that to keep our research into STSs proceeding forward, we needed to perform additional studies with a clinically relevant HDAC inhibitor; entinostat.

Pazopanib interacted with entinostat at clinically relevant concentrations to kill a genetically wide variety of sarcoma cell types. Cells exposed to the drug combination exhibited compensatory activation of receptor and non-receptor tyrosine kinases in parallel with mTOR inactivation and phosphorylation of autophagosome regulatory proteins that collectively will enhance autophagosome formation. The drug combination reduced the expression and activity of GRP78, HSP90, and HSP70 that correlated with prolonged endoplasmic reticulum stress signaling. Prolonged ER stress signaling will lead to reduced expression of multiple proteins that have short half-lives, such as MCL-1 and BCL-XL. Based on these findings we predicted that inhibition of ERBB1/2/4 using neratinib or inhibition of c-MET using crizotinib would block compensatory survival signaling and enhance the anti-tumor efficacy of [pazopanib + entinostat]. Both neratinib and crizotinib were capable of significantly enhancing [pazopanib + entinostat] lethality. Neratinib, in addition to being an inhibitor of ERBB family tyrosine kinases, is also an inhibitor of Sterile 20 (Ste20) serine/threonine kinases. One outcome of this unexpected neratinib biology was that the drug regulated the Hippo Pathway. Coordinated signaling by RAS proteins and the co-transcriptional regulators YAP and TAZ has been shown to promote tumor growth, invasion and chemotherapy resistance (23–26). Individually, neratinib as well as [pazopanib + entinostat] exposure increased the phosphorylation of YAP and TAZ and interacted together to further enhance phosphorylation of the co-transcription factors. Knock down of YAP enhanced the lethality of both the two-drug and three-drug combinations.

The mechanism by which neratinib enhanced [pazopanib + entinostat] lethality is multi-factorial. Exposure of sarcoma cells to [pazopanib + entinostat] caused activation of ERBB1, ERBB2, c-KIT, c-MET, and c-SRC. Neratinib, to a greater extent than crizotinib, prevented or significantly reduced the activation of ERBB1, ERBB2, c-KIT, c-MET, and c-SRC. Neratinib, and to a lesser extent crizotinib, facilitated the down-regulation of K-RAS and of N-RAS (6). Exposure of sarcoma cells to [pazopanib + entinostat] caused activation of ATM, AMPK, and ULK-1 S317 phosphorylation. Neratinib, to a greater extent than crizotinib, enhanced ATM activation and ULK-1 S317 phosphorylation, whereas the activation of AMPK was enhanced to a similar extent by both RTK inhibitors. Exposure of cells to [pazopanib + entinostat] reduced the phosphorylation of ULK-1 S757 that was associated with reduced mTORC1 and mTORC2 activities. Both crizotinib and neratinib acted to cause further mTOR inactivation and further ULK-1 S757 dephosphorylation. The inactivation of AKT was more effectively caused by [pazopanib + entinostat] combinations with neratinib than crizotinib, though the dephosphorylation of p70 S6K T389 was similar whether using neratinib or crizotinib. Neratinib, compared to crizotinib, was more effective at promoting inactivation of STAT3 and STAT5. Neratinib, compared to crizotinib, was more effective at causing increased expression of Beclin1 and ATG5. Both neratinib and crizotinib acted to facilitate reduced expression of MCL-1 and BCL-XL.

We have previously published that treatment of cells with either [pazopanib + HDAC inhibitor] or [neratinib + HDAC inhibitor] increases the levels of autophagosomes and autolysosomes in tumor cells. More recently, we have shown that neratinib is capable of inducing LC3-associated phagocytosis, which requires the protein Rubicon (10). And, compared to other autophagy-regulatory proteins whose expression we have manipulated, e.g., Beclin1, ATG5, knock down of Rubicon was significantly more protective. In our sarcoma studies we also discovered that Rubicon knock down was significantly more protective against neratinib, [pazopanib + entinostat] and [pazopanib + entinostat + neratinib] when compared to Beclin1/ATG5. Although there are no published studies discussing LAP in sarcomas, significant literature exists defining the role of autophagy in the biology of sarcomas (23–26). For example, over-expression of Beclin1 can promote autophagy-dependent sarcoma growth and resistance to chemotherapy. Combined inhibition of HDACs and BET bromodomains can facilitate myoblastoma/sarcoma cell differentiation, which requires phosphorylation, i.e., inactivation and cytoplasmic localization, of the Hippo Pathway effector YAP. Furthermore, this differentiation process required increased signaling from unfolded protein response effectors, i.e., phosphorylation of eIF2α S51. As part of what must be a feedback regulatory mechanism in the myoblastoma cells, it was also shown that dephosphorylated YAP, i.e., activated and nuclear localized, could act to suppress activation of PERK/the unfolded protein response. Knock down of YAP promoted LC3A/B conversion and expression of Beclin1 and ATG13, collectively arguing that autophagosome formation was being enhanced.

In our prior studies we have linked elevated UPR signaling/eIF2α S51 phosphorylation to mTOR dephosphorylation and increased autophagosome formation, with autophagy acting to preserve cell viability (6). Exposure of sarcoma cells to neratinib, [pazopanib + entinostat], or the three-drug combination enhanced YAP and TAZ phosphorylation in parallel to a profound inactivation of mTOR resulting in phosphorylation of gate-keeper protein ATG13 S318. Knock down of eIF2α maintained MCL-1 and BCL-XL expression and blunted the drug-induced inactivation of mTOR but did not prevent YAP phosphorylation. Separately, knock down of Rubicon did not prevent the declines in MCL-1 and BCL-XL expression but did prevent the phosphorylation of YAP and the dephosphorylation of mTOR. Collectively, the data strongly argue that coordinated signaling from LAP [neratinib] and from the UPR [pazopanib + entinostat] play separate and overlapping roles in their ultimate downstream effect of killing sarcoma cells.

It is known that sarcoma cells utilize ERBB1 to promote anoikis resistance (27–29). Thus, a three-drug combination of [pazopanib + entinostat + neratinib] has the potential to considerably reduce the levels of circulating tumor cells, as well as those localized and growing within tissues. In addition, as neratinib blocks both ERBB2 and Ezrin phosphorylation, this drug may have particular utility in sarcomas (29). Previously we have demonstrated that pazopanib can be combined with the HDAC inhibitors sodium valproate or AR42 to kill a variety of tumor cell types growing *in vivo* (5). The present studies demonstrated that pazopanib can be combined with another HDAC inhibitor, entinostat, to suppress tumor growth (6). Neratinib as a single agent had a stronger anti-tumor effect compared to pazopanib; neratinib and entinostat also interacted to suppress tumor growth. The three-drug combination of [pazopanib + entinostat + neratinib], modestly but significantly suppressed tumor volumes below those of [pazopanib + entinostat] or [neratinib + entinostat]. Collectively, the *in vitro* and *in vivo* findings from our study support performing a new clinical trial in STSs combining pazopanib and entinostat. Although further pre-clinical studies are needed, our data also further support our open phase I trial combining neratinib and entinostat in all solid tumor patients (NCT03919292), and our proposed pre-clinical studies in uveal melanoma using the [neratinib + entinostat] combination.

## Data Availability

All datasets generated for this study are included in the manuscript/Supplementary Files.

## Ethics Statement

The animal study was reviewed and approved by Virginia Commonwealth University IACUC.

## Author Contributions

LB and JR performed the studies and assisted PD in producing graphs. PD and AP wrote the manuscript.

### Conflict of Interest Statement

The authors declare that the research was conducted in the absence of any commercial or financial relationships that could be construed as a potential conflict of interest.

## Abbreviations

AIF: apoptosis inducing factor
AMPK: AMP-dependent protein kinase
ca: constitutively active
CMV: empty vector plasmid or virus
dn: dominant negative
ENT: entinostat
ER: endoplasmic reticulum
ERK: extracellular regulated kinase
HDAC: histone deacetylase
IP: immunoprecipitation
JAK: Janus Kinase
MAPK: mitogen activated protein kinase
mTOR: mammalian target of rapamycin
NER: neratinib
PAZ: pazopanib
PI3K: phosphatidyl inositol 3 kinase
PTEN: phosphatase and tensin homolog on chromosome ten
ROS: reactive oxygen species
SCR: scrambled
si: small interfering
STAT: Signal Transducers and Activators of Transcription
VAL: sodium valproate
VEH: vehicle control.
