# Supplementary material for: The Lethality of [Pazopanib + HDAC Inhibitors] Is Enhanced by Neratinib
Source: Front Oncol. 2019 Jul 18;9:650. doi: 10.3389/fonc.2019.00650 (PMC6657367; doi:10.3389/fonc.2019.00650)
Supplement: Supplementary file 1 [file Data_Sheet_1.PDF]

**Figure S1. Neratinib, and when combined with [pazopanib + entinostat] reduces MST1, MST3 and MST4 activity in HT1080 cells.** Human sarcoma cells were treated with vehicle control, [pazopanib (P, 1.0 μM) + entinostat (E, 50 nM)], neratinib (N, 50 nM) or the drugs as indicated in combination for 4h and for 8h. At each time point cells were fixed in place and immunofluorescence staining performed to detect the expression and phosphorylation of the indicated proteins. The percentage change in fluorescence intensity compared to vehicle control (defined as 100%) is plotted (n = 3 +/-SEM). * $p < 0.05$ less than [P+E] value; # $p < 0.05$ greater than [P+E] value; ¶ $p < 0.05$ less than vehicle control value; £ $p < 0.05$ greater than vehicle control value.

**Figure S2. Neratinib, and when combined with [pazopanib + entinostat] reduces MST1, MST3 and MST4 activity in MES cells.** Human sarcoma cells were treated with vehicle control, [pazopanib (P, 1.0 μM) + entinostat (E, 50 nM)], neratinib (N, 50 nM) or the drugs as indicated in combination for 4h and for 8h. At each time point cells were fixed in place and immunofluorescence staining performed to detect the expression and phosphorylation of the indicated proteins. The percentage change in fluorescence intensity compared to vehicle control (defined as 100%) is plotted (n = 3 +/- SEM). * $p < 0.05$ less than [P+E] value; # $p < 0.05$ greater than [P+E] value; ¶ $p < 0.05$ less than vehicle control value; £ $p < 0.05$ greater than vehicle control value.

S1

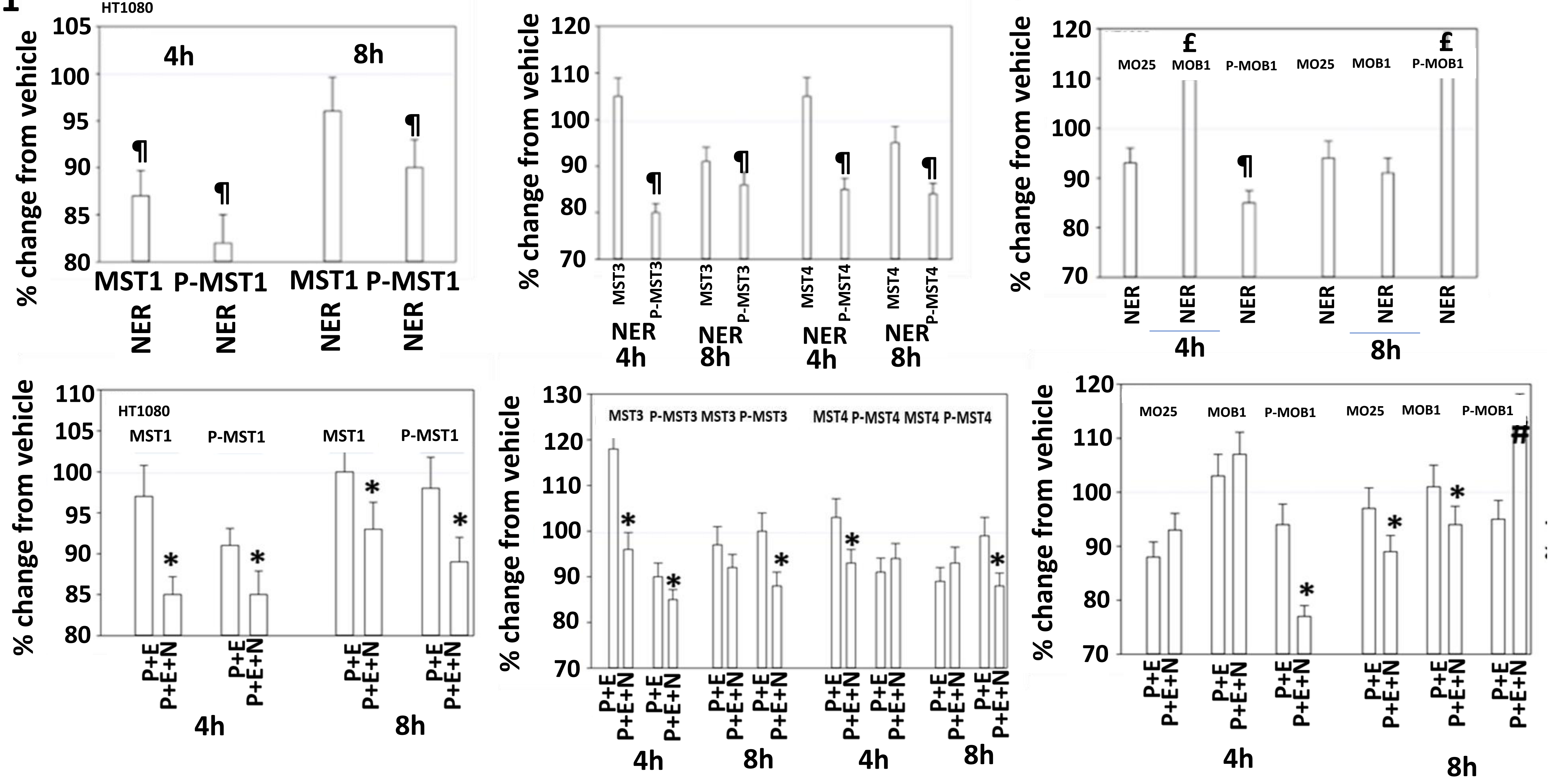
HT1080
4h
8h
% change from vehicle
MST1 P-MST1 MST1 P-MST1
NER
MST3 P-MST3 MST3 P-MST3 MST4 P-MST4 MST4 P-MST4
MO25 MOB1 P-MOB1 MO25 MOB1 P-MOB1
P+E P+E+N

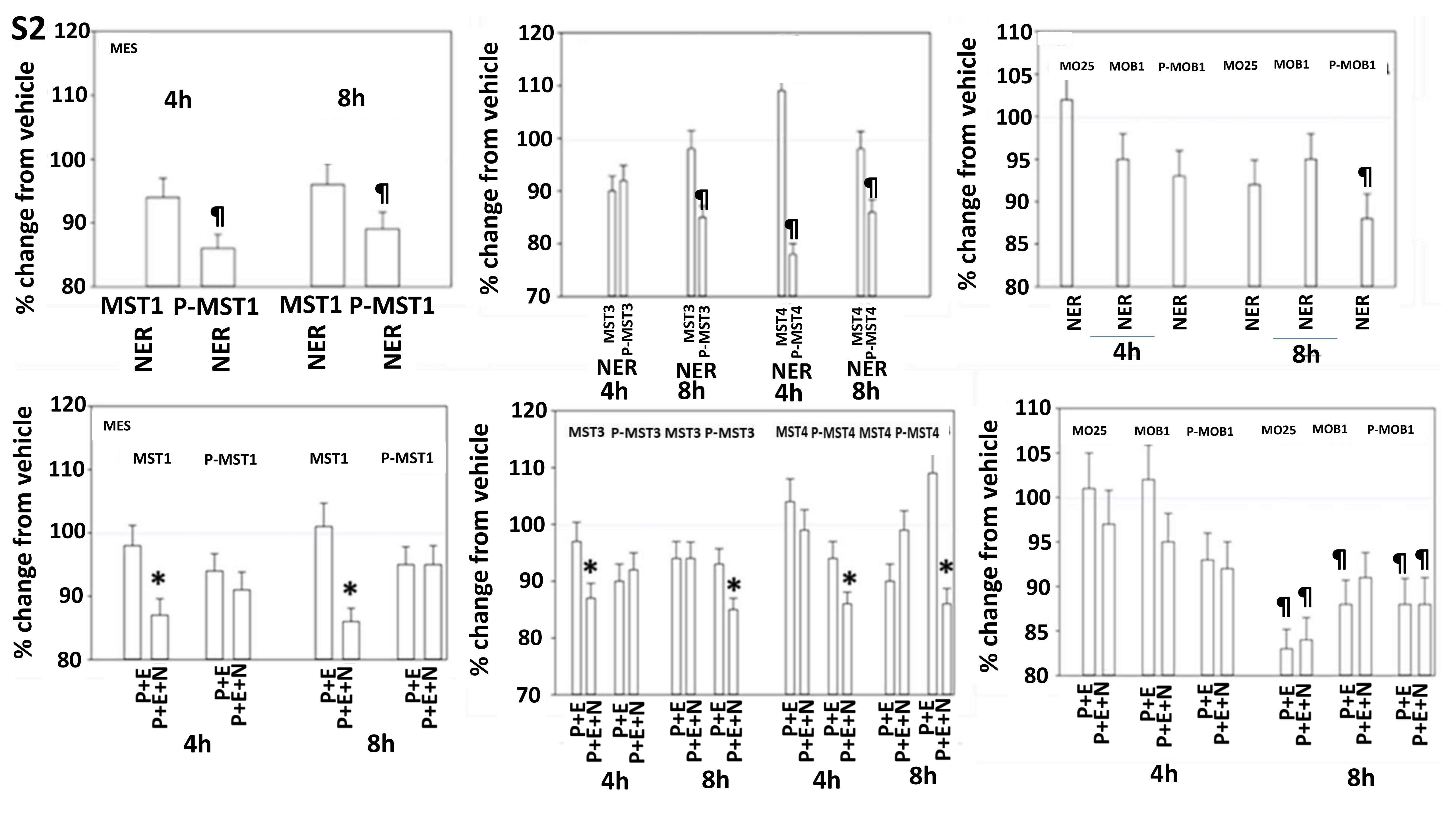
S2
MES
4h
8h
MST1
P-MST1
NER
% change from vehicle
MST3
P-MST3
MST4
P-MST4
MO25
MOB1
P-MOB1
P+E
P+E+N
¶
*
